# Perigraft air is not always pathological: a case report

**DOI:** 10.1186/1752-1947-1-63

**Published:** 2007-08-12

**Authors:** Elizabeth Ball, Gareth Morris-Stiff, Mari Coxon, Michael H Lewis

**Affiliations:** 1 Department of Surgery, Royal Glamorgan Hospital, Ynysmaerdy, Llantrisant, UK

## Abstract

**Background:**

The presence of perigraft air is a common finding in the immediate post-operative phase following abdominal aortic aneurysm repair whilst the later appearance of air, in association with elevated inflammatory markers, is regarded as being indicative of the serious complication of graft infection. What is not known is at what timepoint following surgery does the perigraft air become a significant finding.

**Case Presentation:**

We report the case of a 71 year old man who underwent a computed tomography scan 15 days following repair of an abdominal aortic aneurysm because of the presence of unexplained pyrexia. The scan showed the presence of perigraft air and a small haematoma. The patient was managed conservatively and after 6 weeks the air and haematoma had resolved completely.

**Conclusion:**

The presence of perigraft air in the early postoperative phase is probably a normal finding, is not associated with graft infection and can be managed non-operatively.

## Background

Intra-abdominal free gas is a normal finding in the immediate postoperative period following a laparotomy. Studies evaluating the role of computerised tomography (CT) scanning in the early postoperative period (within 7 days of surgery) following aortic aneurysm repair have similarly shown that periprosthetic air is a not uncommon finding and simply represents air trapped in the tissue planes between the graft and the aneurysm sac [1].

However, periprosthetic gas later in the postoperative period following abdominal aortic aneurysm repair is not such a benign finding and is said to be a reliable indicator of graft infection. This complication is associated with a mortality rate of 25–75% [1,2]. Few investigators have looked at the early postoperative period following resolution of the 'laparotomy' air.

## Case presentation

A 71 year old retired driver presented to the vascular clinic with a calf claudication distance of three hundred yards. He also complained of rest pain in his toes. He had been a non-smoker for thirteen years. His risk factors for peripheral vascular disease included diet-controlled diabetes and hypertension. He had suffered a left-sided stroke fifteen years prior to admission, from which he had made a full recovery. He was taking regular aspirin, allopurinol and an oral hypoglycaemic.

Examination of his abdomen revealed a tender pulsatile epigastric mass. All his peripheral pulses were present and there were no bruits in either his femoral arteries or adductor canals. An ultrasound scan confirmed the presence of an abdominal aortic aneurysm measuring 5.2 × 5.5 cm, with normal calibre iliac vessels. A duplex scan showed a fifty percent stenosis in the mid-portion of the left superficial femoral artery. Because of the tenderness, the patient was admitted and underwent elective repair of his aneurysm using a woven polyester graft. The procedure was uncomplicated and no haemostatic agents were used.

On the fifth post-operative day he developed pyrexia of 38°C. His white cell count at that time was 9.2 × 10^9^/l. Clinical examination was unremarkable. Urine, blood and wound cultures were sterile and a chest radiograph was unremarkable.

On the eighth post-operative day the patient complained of pain and loss of feeling in his left leg. On examination his foot was cool, and pedal pulses were impalpable. A duplex scan showed that the left superficial femoral artery was occluded throughout its length. The patient was taken to theatre for an emergency left femoral embolectomy. The procedure was successful, he made a good recovery and regained both sensation and movement in his leg.

**Figure 1:**
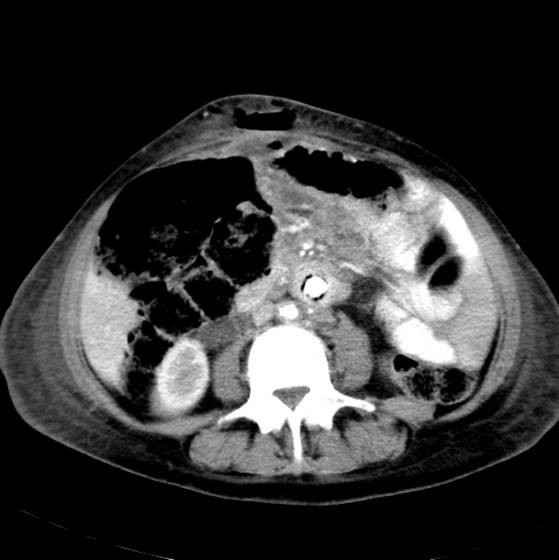
CT scan on 15^th ^postoperative day demonstrating a rim of air around the graft.

Over the course of the subsequent week, the patient continued to exhibit a mild pyrexia despite absence of symptoms of graft infection such as malaise or back pain, and he did not experience any gastrointestinal tract bleeding. Furthermore, inflammatory markers including full blood count, C-reactive protein and erythrocyte sedimentation rate were normal and all cultures were sterile. A computed tomography scan on the fifteenth post-operative day using both intravenous and oral contrast (Figure 1) showed a cuff of abnormal soft tissue, consistent with a small perigraft haematoma, with gas bubbles surrounding the lower end of the graft.

He was closely observed for a further week. Repeat blood cultures were negative, and inflammatory markers remained within normal limits. As the patient was now apyrexial and continued to be asymptomatic, the decision was made to discharge the patient. A repeat CT scan performed six weeks following his aortic surgery showed that the perigraft air had completely resolved and the haematoma had organised. The patient has been followed up for two year post-operatively and remains asymptomatic.

## Discussion

Graft infection is a recognised but catastrophic complication of aortic bypass surgery, with mortality between 25 and 75% [1,3]. The corrective treatment also carries a high morbidity and mortality. Graft sepsis can be difficult to identify in the early post-operative period. The clinical presentation may be straightforward. However it can also present with non-specific symptoms such as malaise, back pain and fever. With such a high mortality it is vital to diagnose this potentially life-threatening condition as quickly as possible and CT is the imaging modality of choice.

There is very little data detailing the natural history of periprosthetic air in the early post-operative period. Two prospective studies have been performed with similar results. Qvardfordt *et al*. [2] studied 29 patients who underwent reconstructive aortic surgery, performing a CT scan at 7, 48 and 102 days post-operatively. Only 4 patients had perigraft air at 7 days, and this air had completely resolved by day 28. O'Hara *et al*. looked at 26 patients, scanning them on days 3, 7 and 52. Seventeen patients had perigraft air on day 3, and seven on day 7. No patient had residual perigraft air on the final scan.

There is however no data regarding perigraft air in the period 2–4 weeks following abdominal aortic surgery such as in our case. O'Hara *et al*. found that patients with larger aneurysms (especially over 6 cm) have a higher incidence of perigraft air being detected on an early CT scan. Our patient had loculated perigraft air on a CT scan performed two weeks after surgery which had resolved by 6 weeks.

In this case, as the inflammatory markers were normal, it is likely that the air identified on the CT scan simply represented air remaining following the initial repair that had not completely resolved. Another option is that this may have been indicative of a subclinical infection although this is less likely as no infection has become evident during 2 years of follow-up and there were no other signs of graft infection such as: persisting perigraft fluid; or pseudoaneurysm [1,4,5]. There was a little perigraft soft tissue attenuation which was believed to be due to a resolving haematoma in keeping with the recent surgery. In addition there were no signs associated with the presence of an aortoenteric fistula such as focal bowel wall thickening or paraprosthetic extravasation of enteric contrast or of intravenous contrast.

Had there been systemic evidence of infection then additional radiological investigations, in particular isotope studies such as indium-111 white blood cell, gallium-67 citrate, or Tc-99m hexametazime scanning could have been performed to try and identify perigraft infection [1].

We suggest that there is a need for further studies to accurately record the natural history of perigraft air in the first month following surgery, as not all cases may represent infected grafts. The question arises as to how often you repeat a CT scan having found post-operative perigraft air, and whether the finding of perigraft air with non-specific clinical symptoms indicates early graft infection, or a normal stage in the healing process.

## Competing interests

The author(s) declare that they have no competing interests.

## Authors' contributions

All authors have read and approved the final manuscript.
